# On the data to know the prioritization and vulnerability of patients on surgical waiting lists

**DOI:** 10.1016/j.dib.2020.105310

**Published:** 2020-02-22

**Authors:** Fabián Silva-Aravena, Eduardo Álvarez-Miranda, César A. Astudillo, Luis González-Martínez, José G. Ledezma

**Affiliations:** a Departamento de Planificación y Control de Gestión, Hospital Regional de Talca, Chile; b D.Sc. Program in Systems Engineering, Faculty of Engineering, Universidad de Talca, Campus Curicó, Chile; c Department of Industrial Engineering, Faculty of Engineering, Universidad de Talca, Curicó, Chile; d Instituto Sistemas Complejos de Ingeniería (ISCI), Chile; e Departamento de Ciencias de la Computación, Universidad de Talca, Curicó, Chile; f Servicio de Otorrinolaringología, Hospital Regional de Talca, Chile

**Keywords:** Waiting list, Biopsichosocial criteria, Priorization, Vulnerability, Medical decision making

## Abstract

The data presented in this article are complementary material to our work entitled “A decision support system for prioritization of patients on surgical waiting lists: A biopsychosocial approach”. We prepared, together with physicians, a survey was used in the otorhinolaryngology unit of the Hospital of Talca for a period of five months, between February 05, 2018 and June 29, 2018. Two hundred and five surveys were collected through 20 biopsychosocial criteria, which allowed measuring the priority and vulnerability of patients on the surgical waiting list. The data allow choosing and preparing patients for surgery according to both a dynamic score and a vulnerability level.

**Table:** Specifications Table

Subject	Health.
Specific subject area	Priority and vulnerability of patients.
Type of data	Tables, figures.
How data were acquired	During the patient's interview, physicians fill a survey that indicates different aspects regarding the condition of the patient. This data is later used for estimating the priority and vulnerability of the surgical waiting list.
Data format	Raw and analysed.
Parameters for data collection	The construction of the dataset was done for analysis. The specialists in otolaryngology suggested the process of data collection in the application of their day-to-day activities with patients.
Description of data collection	The data of surveys were collected in the polyclinic of the otorhinolaryngology unit of the Hospital of Talca for a period of five months, between February 05, 2018 and June 29, 2018. The process of starting the patient's survey was carried out at first by a nurse and then finished by a physician. Then, to prioritize and measure the vulnerability of patients on the surgical waiting list of the otorhinolaryngology unit, we propose a method of data analysis. Specifically, we transform physicians’ knowledge into quantitative information that allowed us to; (1) characterize patients and (2) create a method that can rank patients according to two dynamic measures, the score and vulnerability. Data analysis allows the development of strategic actions to optimize the management of the surgical waiting list.
Data source location	Institution: Hospital of Talca. City/Town/Region: Talca. Country: Chile. Latitude and longitude (and GPS coordinates) for collected samples/data: Latitude: −35.426971; Longitude: −71.646647
Data accessibility	The raw data files are provided in the following: Repository name: Google Drive. Data identification number: 05122019 Direct URL to data: https://drive.google.com/file/d/1iBqeFkiE27fbYXI7hqRXyw9h4nMgCBxc/view?usp=sharing All other data is with this article.
Related research article	Authors: Fabián Silva-Aravena, Eduardo Álvarez-Miranda, César A. Astudillo, Luis González-Martínez, José G. Ledezma. Title: A decision support system for prioritization of patients on surgical waiting lists: A biopsychosocial approach. Journal: Submitted to *Annals of Operational Research.*

**Table:** 

**Value of the Data** • The data contain records associated to 20 biopsychosocial parameters from 205 patients. These data (i) include the opinion of seven physicians, (ii) allow the characterization of the patients, (iii) allow the elaboration of a priority score, and (iv) obtain the vulnerability measure of the patients on the surgical waiting list of the otorhinolaryngology unit. • Using data analysis techniques, we propose strategic actions for ranking patients. This methodology could be useful for optimizing decision-making. • The data allow the design of strategic actions for policy makers in the health care system. • The data set can be used by other researchers to carry out studies in the area of patients on waiting lists.

## 1 Data description

**Fig. 1:**
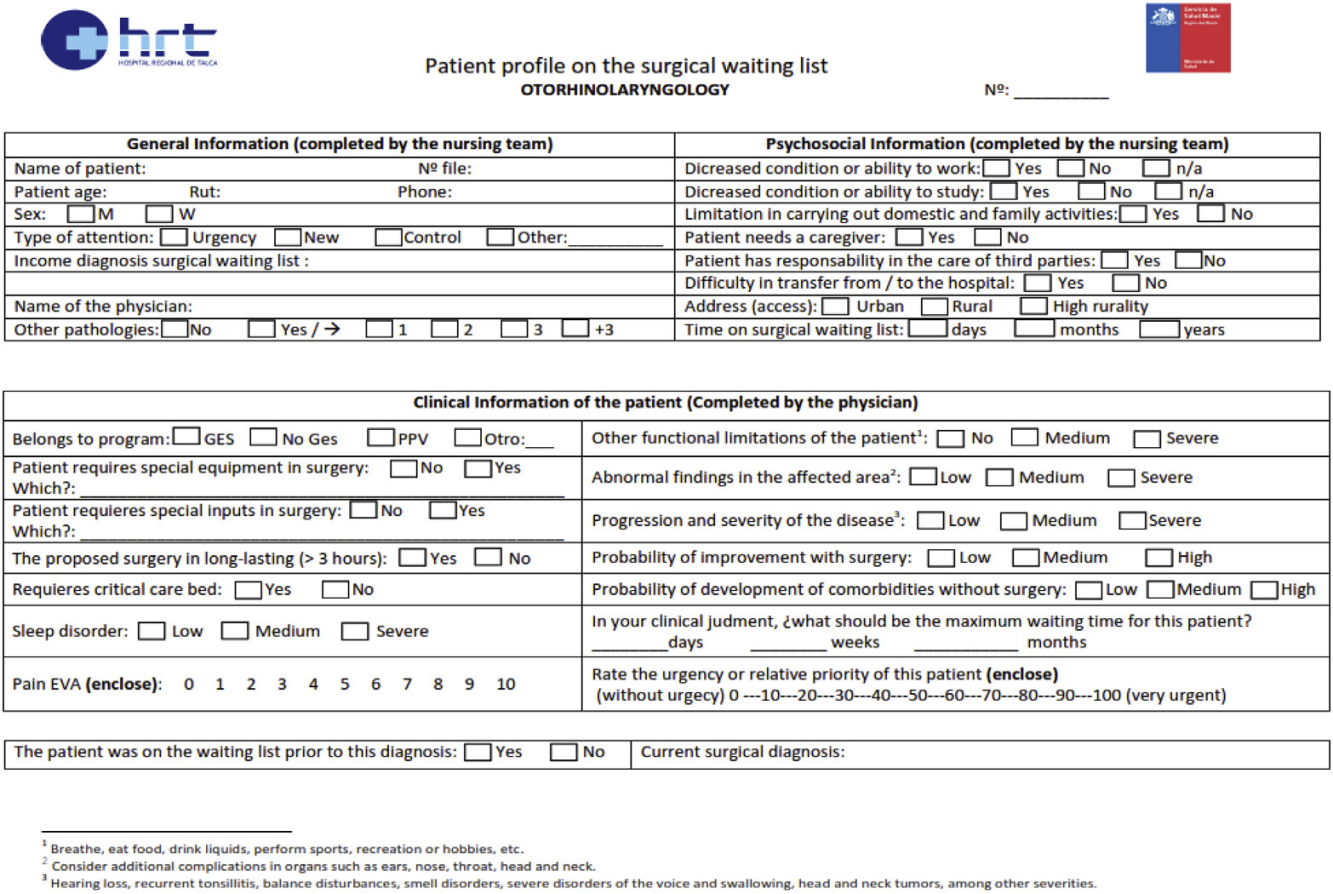
Survey to measure the prioritization of otorhinolaryngology patients in the Hospital of Talca, Chile.

**Fig. 2:**
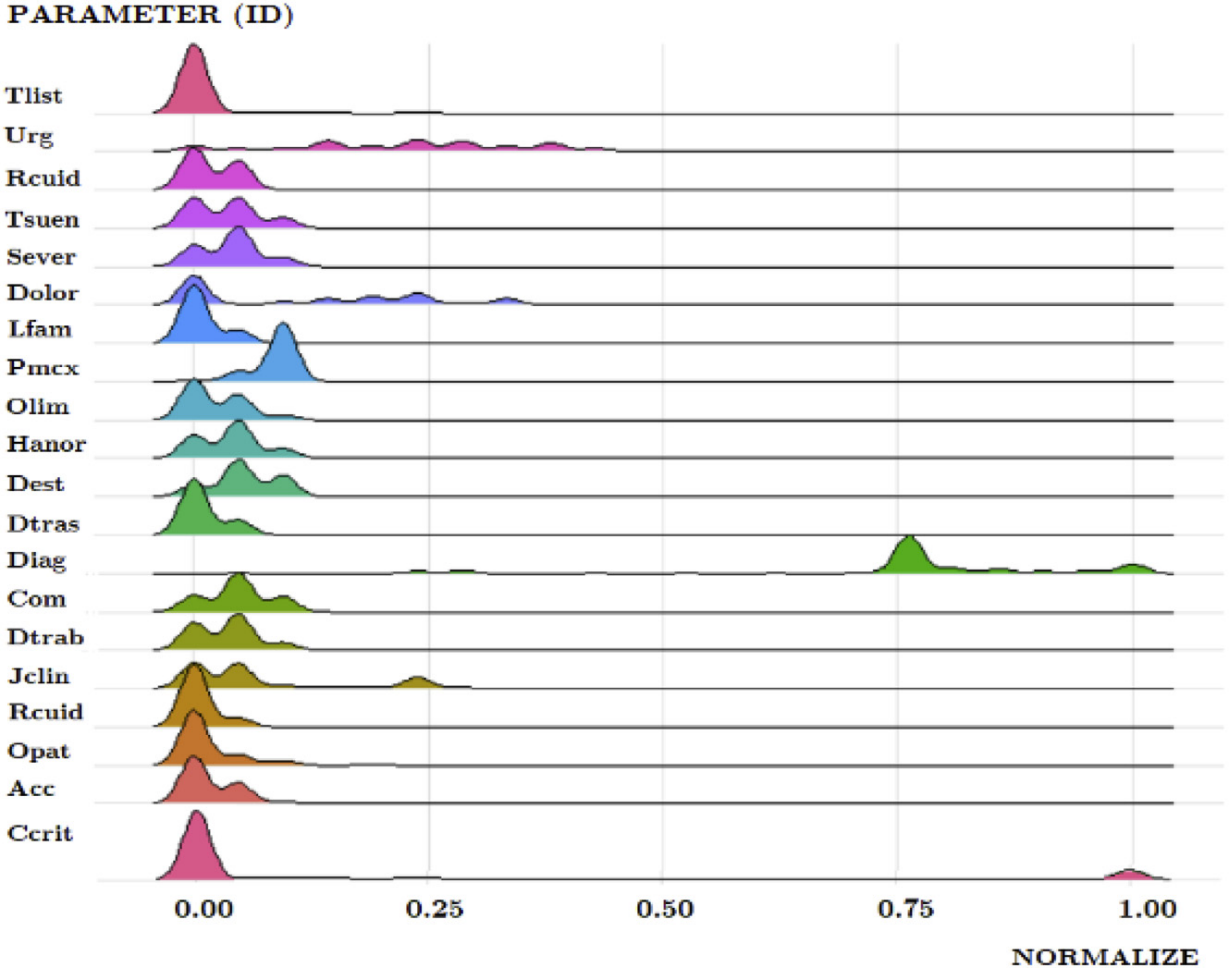
Data distribution for each biopsychosocial parameter. For the sake of clarity, the ranges have been normalized.

The data set in this article describes biopsychosocial parameters of the patients of the otolaryngology unit on the surgical waiting list. Fig. 1 describes in detail the survey applied to each of the patients who consulted for their disease in the physician's office. With this instrument, nurses and physicians collected the data that would then be analysed to prioritize their patients. Fig. 2 describes the normalization of the parameters, which were transformed before data analysis. Table 1 describes the opinion of physicians concerning patient ages. Table 2 shows central tendency measures of the 20 parameters. Table 3 describes the parameters and how they were incorporated into the data set. Table 4 describes the opinion given to each parameter by each of the physicians. Table 5, Table 6, Table 7 describe the degree of importance granted by the physicians to each of the variables. Finally, Table 8 list the diagnoses more frequently of otorhinolaryngology unit in Hospital of Talca.

**Table 1:** Opinions of the physicians regarding priority by age group.

Types	Age group (years)	P1	P2	P3	P4	P5	P6	P7
Infant	0–1	1	3	5	6	6	5	6
Child	2–12	2	6	6	6	6	6	5
Teenager	13–18	3	6	4	6	6	4	4
Young adult	19–40	4	5	1	6	6	3	3
Adult	41–59	5	4	2	6	6	2	2
Elderly	greater than 60	6	1	3	6	6	1	1

**Table 2:** Measures of central tendency of the considered biopsychosocial parameters.

Parameters	Min.	1st Qu.	Median	Mean	3rd Qu.	Max.	Mode
Sever	0	0	1	0.9024	1	2	1
Urg	0	3	5	4.9020	7	9	3
Jclin	1	4	6	5.9020	7	9	6
Tsuen	0	0	1	0.7854	1	2	0
Tlist	0	0	0	0.1415	0	8	0
Pmcx	0	2	2	1.8100	2	2	2
Dest	0	0	1	0.9756	1	2	1
Com	0	1	1	0.9951	1	2	1
Lfam	0	0	0	0.1805	0	1	0
Hanor	0	0	1	0.8829	1	2	1
Opat	0	0	0	0.3707	1	4	0
Diag	0	11	11	10.36	13	17	11
Olim	0	0	0	0.4488	1	2	0
Ncuid	0	0	0	0.3707	1	1	0
Rcuid	0	0	0	0.1122	0	1	0
Dolor	0	0	2	2.6730	5	8	0
Dtrab	0	0	1	0.7805	1	2	1
Acc	0	0	0	0.3463	1	2	0
Dtras	0	0	0	0.2146	0	1	0
Ccrit	0	0	0	0.0098	0	1	0

**Table 3:** Description of the biopsychosocial parameters presented in the dataset.

ID	Name	Type	Values	In dataset
Sever	Progression and severity of the disease	Ordinal	low/medium/high	{0, 1, 2}
Urg	Urgency	Percentage	{0%, …, 100%}	{0, …, 10}
Jclin	Clinical judgment maximum wait time (month)	Numeric	{0, …, 60}	{0, …, 9}
Tsuen	Sleep disorder	Ordinal	low/medium/severe	{0, 1, 2}
Tlist	Time on the surgical waiting list (month)	Numeric	{0, …, 60}	{0, …, 9}
Pmcx	Probability of improvement with surgery	Ordinal	low/medium/high	{0, 1, 2}
Dest	Diminished capacity of study	Ordinal	NA/No/Yes	{0, 1, 2}
Com	Probability of developing comorbidities without surgery	Ordinal	low/medium/high	{0, 1, 2}
Lfam	Family activities	Ordinal	No/Yes	{0, 1}
Hanor	Affected area	Ordinal	low/medium/severe	{0, 1, 2}
Opat	Other additional pathologies	Numeric	{0, 1, 2, 3, +3}	{0, …, 4}
Diag	Diagnosis of admission to the waiting list	Numeric	{0, …, 17}	{0, …, 17}
Olim	Other functional limitations	Ordinal	no/medium/severe	{0, 1, 2}
Ncuid	The patient needs a caregiver	Ordinal	No/Yes	{0, 1}
Rcuid	Responsibility in caring for another person	Ordinal	No/Yes	{0, 1}
Dolor	EVA scale pain	Numeric	{0, …, 10}	{0, …, 10}
Dtrab	Working capacity	Ordinal	NA/No/Yes	{0, 1, 2}
Acc	Access	Ordinal	urban/rural/high rurality	{0, 1, 2}
Dtras	Difficulty in transferring from/to the hospital	Ordinal	No/Yes	{0, 1}
Ccrit	Need for critical beds	Ordinal	No/Yes	{0, 1}

**Table 4:** Scores assigned by the seven physicians to the biopsychosocial parameters.

ID	P1	P2	P3	P4	P5	P6	P7	Total	$w_{i}$(%)	Rank
Sever	10	10	10	9	10	10	10	69	8.1	1
Urg	8	8	10	10	10	9	10	65	7.6	2
Jclin	3	8	8	10	8	9	10	56	6.6	3
Tsuen	6	10	7	7	6	10	8	54	6.3	4
Tlist	7	9	5	9	9	6	8	53	6.2	5
Pmcx	1	10	8	9	4	10	5	47	5.5	6
Dest	5	8	8	8	4	6	7	46	5.4	7
Com	1	10	7	7	5	9	6	45	5.3	8
Lfam	5	8	8	7	4	6	7	45	5.3	9
Hanor	2	6	7	8	5	10	6	44	5.2	10
Opat	2	7	6	7	4	9	5	40	4.7	11
Diag	2	9	9	2	5	6	6	39	4.6	12
Olim	2	7	4	7	5	3	10	38	4.5	13
Ncuid	5	9	6	4	5	1	7	37	4.3	14
Rcuid	5	10	5	8	5	1	3	37	4.3	15
Dolor	1	4	4	7	5	3	10	34	4.0	16
Dtrab	5	8	8	5	4	1	1	32	3.8	17
Acc	5	7	4	1	2	6	3	28	3.3	18
Dtras	5	7	3	1	4	1	3	24	2.8	19
Ccrit	1	8	6	1	2	1	1	20	2.3	20
Total	–	–	–	–	–	–	–	853	100.0	-

**Table 5:** Opinion of physicians for each of the variables. $\alpha_{i,p}$.

α*i*,*p*	P1	P2	P3	P4	P5	P6	P7	Total	Weight%
Sever = low	1	0	3	0	1	1	1	7	6.5%
Sever = medium	5	0	6	5	5	5	5	31	28.7%
Sever = high	10	10	10	10	10	10	10	70	64.8%
Urg = 0%	0	0	0	0	0	0	0	0	0.0%
Urg = 10%	1	0	1	1	1	1	1	6	1.6%
Urg = 20%	2	0	2	2	2	1	2	11	2.9%
Urg = 30%	3	0	3	3	3	1	3	16	4.3%
Urg = 40%	4	0	4	4	4	5	4	25	6.7%
Urg = 50%	5	5	5	5	5	5	5	35	9.4%
Urg = 60%	6	6	6	6	6	5	6	41	11.0%
Urg = 70%	7	7	7	7	7	5	7	47	12.6%
Urg = 80%	8	8	8	8	8	10	8	58	15.5%
Urg = 90%	9	9	9	9	9	10	9	64	17.2%
Urg = 100%	10	10	10	10	10	10	10	70	18.8%
Jclin => 60 (months)	0	2	0	1	1	6	0	10	2.8%
Jclin = 49 − 60	0	4	2	2	2	6	0	16	4.5%
Jclin = 37 − 48	0	4	3	3	3	6	0	19	5.4%
Jclin = 25 − 36	0	5	3	4	4	6	0	22	6.2%
Jclin = 19 − 24	3	5	4	5	5	6	0	28	7.9%
Jclin = 13 − 18	4	6	4	6	6	6	0	32	9.0%
Jclin = 10 − 12	5	6	5	7	7	7	6	43	12.1%
Jclin = 7–9	6	10	7	8	8	8	6	53	14.9%
Jclin = 4 − 6	7	10	8	9	9	9	10	62	17.5%
Jclin = 0 − 3	10	10	10	10	10	10	10	70	19.7%
Tsuen = low	1	0	1	0	1	1	1	5	5.0%
Tsuen = medium	5	0	4	5	5	5	5	29	29.0%
Tsuen = severe	8	10	8	10	10	10	10	66	66.0%
Tlist = 0 – 3 (months)	0	4	0	5	1	1	5	16	3.6%
Tlist = 4 − 6	1	5	0	5	2	5	5	23	5.2%
Tlist = 7 − 9	2	5	2	5	3	5	10	32	7.2%
Tlist = 10 − 12	3	9	2	5	4	8	10	41	9.2%
Tlist = 13 − 18	4	9	3	6	5	10	10	47	10.6%
Tlist = 19 − 24	5	9	4	6	6	10	10	50	11.2%
Tlist = 25 − 36	6	10	5	6	7	10	10	54	12.1%
Tlist = 37 − 48	7	10	5	7	8	10	10	57	12.8%
Tlist = 49 − 60	8	10	6	8	9	10	10	61	13.7%
Tlist => 60	9	10	6	9	10	10	10	64	14.4%
Pmcx = low	0	0	1	0	1	1	1	4	4.5%
Pmcx = medium	0	3	3	5	5	8	5	29	33.0%
Pmcx = high	0	10	5	10	10	10	10	65	62.5%
Dest = NA	0	0	0	0	0	0	0	0	0.0%
Dest = No	1	1	1	0	0	0	0	3	6.0%
Dest = Yes	5	9	6	5	5	10	7	65	94.0%

**Table 6:** Opinion of physicians for each of the variables.$\alpha_{i,p}$.

αi,p	P1	P2	P3	P4	P5	P6	P7	Total	Weight%
Com = low	1	0	1	0	1	1	1	5	4.9%
Com = medium	3	7	3	8	5	5	5	36	35.3%
Com = high	5	10	6	10	10	10	10	61	59.8%
Lfam = No	1	2	1	0	0	0	0	4	9.1%
Lfam = Yes	5	8	5	5	5	5	7	40	90.9%
Hanor = low	1	0	1	0	1	1	1	5	5.6%
Hanor = medium	3	6	3	3	5	5	5	30	33.3%
Hanor = severe	5	10	5	5	10	10	10	55	61.1%
Opat = 0	0	2	0	1	1	5	1	10	6.9%
Opat = 1	1	7	1	1	2	9	1	22	15.1%
Opat = 2	3	8	2	1	6	10	1	31	21.2%
Opat = 3	5	9	3	1	8	10	1	37	25.3%
Opat = +3	10	10	4	1	10	10	1	46	31.5%
Diag = 0	10	10	9	8	10	10	10	67	7.6%
Diag = 1	8	10	8	7	9	10	10	62	7.0%
Diag = 2	10	9	9	6	10	8	6	62	7.0%
Diag = 3	7	10	8	7	6	10	10	58	6.5%
Diag = 4	8	9	8	4	8	10	10	57	6.4%
Diag = 5	7	10	8	5	6	9	10	55	6.2%
Diag = 6	7	10	7	4	6	10	10	54	6.1%
Diag = 7	8	10	8	5	9	4	10	54	6.1%
Diag = 8	7	8	7	7	10	4	10	53	6.0%
Diag = 9	7	10	5	7	6	8	10	53	6.0%
Diag = 10	6	7	6	5	6	8	8	46	5.2%
Diag = 11	5	7	7	5	6	5	10	45	5.1%
Diag = 12	5	9	7	6	4	5	5	41	4.6%
Diag = 13	5	5	6	4	6	8	6	40	4.5%
Diag = 14	5	6	6	5	4	5	8	39	4.4%
Diag = 15	5	7	5	4	3	4	8	36	4.1%
Diag = 16	5	5	4	6	2	5	7	34	3.8%
Diag = 17	5	6	3	4	3	5	5	31	3.5%
Olim = No	1	0	1	0	1	1	1	5	4.9%
Olim = medium	3	8	4	5	5	5	5	35	34.0%
Olim = severe	6	10	7	10	10	10	10	63	61.2%
Ncuid = No	1	2	0	0	0	0	0	3	6.8%
Ncuid = Yes	10	8	1	0	5	10	7	41	93.2%
Rcuid = No	1	1	0	0	0	0	0	2	6.1%
Rcuid = Yes	5	9	1	0	5	8	3	31	93.9%

**Table 7:** Opinion of physicians for each of the variables.$\alpha_{i,p}$.

αi,p	P1	P2	P3	P4	P5	P6	P7	Total	Weight%
Dolor = 0	0	0	0	0	0	0	0	0	0.0%
Dolor = 1	1	1	0	1	1	1	1	6	1.6%
Dolor = 2	2	2	1	2	2	1	2	12	3.2%
Dolor = 3	3	3	1	3	3	1	3	17	4.5%
Dolor = 4	4	4	2	4	4	8	4	30	8.0%
Dolor = 5	5	5	3	5	5	8	5	36	9.6%
Dolor = 6	6	6	3	6	6	8	6	41	11.0%
Dolor = 7	7	7	5	7	7	8	7	48	12.8%
Dolor = 8	8	8	5	8	8	10	8	55	14.7%
Dolor = 9	9	9	7	9	9	10	9	62	16.6%
Dolor = 10	10	10	7	10	10	10	10	67	17.9%
Dtrab = NA	0	0	0	0	0	0	0	0	0%
Dtrab = No	1	1	1	0	0	0	0	3	7.1%
Dtrab = Yes	5	9	5	5	5	5	5	39	92.9%
Acc = Urban	1	2	1	0	1	1	1	7	10.5%
Acc = Rural	5	8	1	0	5	1	3	23	34.3%
Acc = High rurality	10	10	1	0	10	1	5	37	55.2%
Dtras = No	1	2	1	0	0	0	0	4	12.5%
Dtras = Yes	5	8	2	0	5	5	3	28	87.5%
Ccrit = No	0	2	1	5	0	0	0	8	34.8%
Ccrit = Yes	0	8	2	5	0	0	0	15	65.2%

**Table 8:** The most frequent diagnoses of otorhinolaryngology patients who enter the surgical waiting list.

Diagnoses
Complicated otitis media
Ear cholesteatoma
Complicated chronic sinusitis
Obstructive tonsil and apnea
Otitis media with efusion
Nasal polyp with apnea
Obstructive sleep apnea
Obstructed lacrimal obstruction
Frontal mucocele
Septodesk with apnea
Simple chronic sinusitis
Hypertrophy of tonsils and adenoids
Recurrent or chronic tonsillitis
Tympanic perforation
Nasal polyp without apnea
Tear duct obstruction
Septo-deviation with apnea
Rinodeviation

## 2 Experimental design, materials, and methods

### 2.1 Construction and completeness of the survey

We prepared, together with physicians, a survey that allows data collection to determine the score and vulnerability measures of patients on the surgical waiting list. The survey was taken in the polyclinic of the otorhinolaryngology unit of the Hospital of Talca for a period of five months, between February 05, 2018 and June 29, 2018. The process of starting the patient's file was carried out at first by a nurse and then finished by a physician. For construction of the dataset, five nurses and seven physicians participated in collecting data. In order to ensure accuracy and consistency of the records, each survey was reviewed by a nurse who verified the patient's medical history and health information system. The survey used can be seen in Fig. 1.

The complete instrument is the one specified in Fig. 1 and is the one used by the clinical team to complete the information. Besides the 20 parameters considered in this work, there are some entries (e.g., “belongs to program”, “does the patient requires special equipment in surgery?“) that are not relevant for our analysis since, as pointed out by physicians, they do not influence the score nor the vulnerability.

Once the survey was completed for each patient who entered the surgical waiting list, we used the individual files for tabulation and subsequent analysis. Then, the data was normalized and prepared for statistical analysis in the same unit of measurement to avoid scaling problems and some outliers were detected. Nevertheless, the raw data were considered as physicians reviewed and validated this information, as well as the specific characteristics of the patients. In Fig. 2, we show the structure of the normalized data distribution from 0 to 1 for each parameter, where we can have a notion of the mean and dispersion of the observations.

### 2.2 Patients description

Table 1 shows the opinion provided by each of the seven physicians when they were consulted about the priority in respect to age group. However, and in subsequent meetings to validate the parameters and prioritization variables, the physicians decided to exclude this parameter since they considered it discriminatory; the same situation occurred with the gender parameter. In summary, we collected 205 surveys, 105 were women and 100 were men. Concerning the ages of the patients, 61.5% of the cases were between 0 and 20 years, 12.5% were between 21 and 40 years, 15.4% between 41 and 60 years, and 10.6% of cases were patients older than 60 years.

### 2.3 Raw data analysis process

Subsequently, to create a prioritization and vulnerability criteria of patients on a surgical waiting list, we processed the raw data and how each of the parameters and biopsychosocial variables that had been measured impacted the patients was discussed with the physicians. To do this, we performed the following steps.

1. We interviewed and consulted each physician looking for relevant parameters and variables that determine the priority of their patients.
2. Each physician quantified each parameter with a score between one and ten, where ten means that the parameter is crucial and a score of one means that the parameter is uninformative.
3. Additionally, physicians scored each parameter.
4. We consolidated raw data of all the opinions of the physicians for analysis.
5. We obtained the average of the opinions of each physician interviewed.

We conduct a review of the literature to know how to prioritize waiting patients in other parts of the world. After that, we met with the physicians and jointly defined the instrument. Then we take the records and perform only descriptive statistics of the data. Also, and for each parameter, we perform the measures of central tendency that can be seen in Table 2.

### 2.4 Description of parameters $w_{i}$

For the experimental design of the survey, we explain in Table 3 each one of the parameters used, considering the (a) ID, (b) name, (c) type, (d) value given by physician, and (e) transformation of their value in the dataset.

Table 4 shows the opinion of the physicians and the value that each of them gave to the 20 parameters. Thus, in order to measure the level of importance of the parameters, they gave a score of 0 to the unimportant ones and up to 10 to the most important ones. To determine the total weight of each parameter, we divided the sum of each parameter's opinions in respect to the sum of all parameters. For example, the parameter *Sever* received 69 points (given by the opinions 10, 10, 10, 9,10, 10, 10, 10). Then, that value was divided by 853 points (which represented the sum of all the parameters). Then, the relative weight of Sever parameter was 8.1%. These weights can be seen in Table 4 and will be used as $w_{i}$ (%).

### 2.5 Description of the variable $\alpha_{i,p}$

Table 3 shows the values of the categorical parameters, which were mapped to numerical values to facilitate the calculation of the score. As a explain before, the $w_{i}$ parameter is common for all patients, but when the physician associates this parameter to a patient with their particular condition, this parameter *Sever* becomes a variable $\alpha_{i,p}$, for example, *Sever* = *low.*

Table 5 shows that the parameter *Sever* was associated to a “*low*” severity with 7 points, a “*medium*” severity with 31 points, and a “*high*” severity with 70 points. Therefore, the relative score of *Sever* = *low* is 7/108·100% = 6.5%, of *Sever* = *medium* is 31/108·100% = 28.7% and of *Sever* = *high* is 70/108·100% = 64.8%. Now, for example, in order to obtain the contribution of *Sever* in the calculation of a patient's score $s_{p}$ with *Sever* = *high*, the relative weight of the parameter *Sever*, which is 8.1%, is multiplied by the relative score associated to *Sever* = *high*, which is 64.8%. The weights of the other variables are shown in Table 5, Table 6, Table 7, and will be used as $\alpha_{i,p}$ to indicate the importance of each of the elements of the parameter $w_{i}$.

### 2.6 Score

The contribution of eachi parameter to the patient score p is given by $z_{i,p}$, which is obtained by multiplying the relative importance of the factor i,$\;w_{i}$, and the value of said factor associated with the patient p, $\alpha_{i,p}$. Finally, we denote as $s_{p}$ to the patient's final score p, which corresponds to the sum of all the $z_{i,p}$, i.e.,

(1) $$s_{p}=\overset{20}{\underset{i=1}{\sum }}z_{i,p}=\;\overset{20}{\underset{i=1}{\sum }}w_{i}\;\alpha_{i,p}$$

### 2.7 Vulnerability

The objective of constructing a measure of vulnerability is to keep patients who might develop comorbidities or increased illness due to waiting more visible on the waiting list. In a similar way with [1], we will construct a way to measure the vulnerability of patients on surgical waiting lists, which we present below;

(2) $$v_{p,t}=\frac{f_{t}-f_{p}}{Jclin_{d,p,t}}$$

where $v_{p,t}$ represents the level of vulnerability of the patient p in the moment t, $f_{t}$ is the moment when the vulnerability is measured,$\;f_{p}$ is the patient's date of admission to the waiting list pand $Jclin_{d,p,t}\;$corresponds to the physician's criteria d in relation to the patient's maximum waiting time p in the moment t. For more details, see [2].

### 2.8 Dynamic prioritization and patient choice

Together with the physicians, we have defined the criteria for dynamic prioritization and patient choice. Dynamic prioritization is built for each diagnosis in Table 8 and how they evolve, and in the patient choice, physicians select patients for surgery while simultaneously assessing their dynamic score and vulnerability measures. For more details of the methodology, see [2].

The proposal makes sense to physicians because the classification system allows them to keep a constant watch on the risk levels of waiting patients. It is for this reason that our proposal focuses on maintaining control of the list through the score and vulnerability of patients.
